# Lactic acid bacteria reduce bacterial diarrhea in rabbits via enhancing immune function and restoring intestinal microbiota homeostasis

**DOI:** 10.1186/s12917-024-03981-5

**Published:** 2024-04-20

**Authors:** Huimin Li, Chaoliang Leng, Nan Chen, Qinchao Ding, Yizhao Yuan, Yilei Zheng, Ge Zhu, Chen Chen, Lichang Xu, Jiangbing Shuai, Qinting Jiang, Daxi Ren, Huanan Wang

**Affiliations:** 1 College of Animal Sciences, Zhejiang University, 310058 Hangzhou, PR China; 2 Henan Provincial Engineering and Technology Center of Animal Disease Diagnosis and Integrated Control, Henan Key Laboratory of Insect Biology in Funiu Mountain, Nanyang Normal University, 473061 Nanyang, PR China; 3 Zhejiang Academy of Science & Technology for Inspection & Quarantine, 310016 Hangzhou, PR China; 4 Zhejiang Jinuo Saibur Biotechnology Co., LTD, 310010 Hangzhou, PR China; 5 Room 515 E Building, 866 Yuhangtang Rd, 310058 Hangzhou, Zhejiang China

**Keywords:** LAB, *Enterococcus faecium*, *Ligilactobaciiius Animalis*, Intestinal flora, Immune function

## Abstract

**Background:**

Numerous previous reports have demonstrated the efficacy of Lactic acid bacteria (LAB) in promoting growth and preventing disease in animals. In this study, *Enterococcus faecium* ZJUIDS-R1 and *Ligilactobaciiius animalis* ZJUIDS-R2 were isolated from the feces of healthy rabbits, and both strains showed good probiotic properties in vitro. Two strains (10^8^CFU/ml/kg/day) were fed to weaned rabbits for 21 days, after which specific bacterial infection was induced to investigate the effects of the strains on bacterial diarrhea in the rabbits.

**Results:**

Our data showed that *Enterococcus faecium* ZJUIDS-R1 and *Ligilactobaciiius animalis* ZJUIDS-R2 interventions reduced the incidence of diarrhea and systemic inflammatory response, alleviated intestinal damage and increased antibody levels in animals. In addition, *Enterococcus faecium* ZJUIDS-R1 restored the flora abundance of *Ruminococcaceae1. Ligilactobaciiius animalis* ZJUIDS-R2 up-regulated the flora abundance of *Adlercreutzia* and *Candidatus Saccharimonas.* Both down-regulated the flora abundance of *Shuttleworthia* and *Barnesiella* to restore intestinal flora balance, thereby increasing intestinal short-chain fatty acid content.

**Conclusions:**

These findings suggest that *Enterococcus faecium* ZJUIDS-R1 and *Ligilactobaciiius animalis* ZJUIDS-R2 were able to improve intestinal immunity, produce organic acids and regulate the balance of intestinal flora to enhance disease resistance and alleviate diarrhea-related diseases in weanling rabbits.

**Supplementary Information:**

The online version contains supplementary material available at 10.1186/s12917-024-03981-5.

## Background

Rabbit farming is becoming a significant emerging industry in developing countries [1]. The most important period in the process of raising rabbits is the weaning period, because infant rabbits are often separated from their mothers and weaned onto solid feed as a replacement for maternal milk. During this period, rabbits are particularly susceptible to gastrointestinal infection due to environmental and physiological changes. These infections are frequently linked to factors such as dietary stress, parasites, and bacterial pathogens [2].

In rabbits farming, several bacterial diseases, such as pathogenic *Escherichia coli*, *Salmonella*, and *Clostridium* species infections, can limit the industry’s development [2, 3]. *Escherichia coli* is the leading cause of bacterial disease, and typically occur as part of a mixed infection with other pathogenic bacteria. Bacterial infection is mainly characterized by frequent bowel movements, diarrhea [4], and the presence of watery or gel-like stools. Therefore, while antibiotics are commonly used to control bacterial infection, they can have toxic side effects that pose a challenge to effective treatment. Antibiotic-associated diarrhea [4] and drug resistance resulting from prolonged antibiotic use have prompted the development of probiotics, among other approaches.

The term ‘probiotic’ was first used in 1965 to describe a substance secreted by an organism that stimulates the growth of other microorganisms [5]. In 2002, probiotic was defined as “living microorganisms that, when consumed in sufficient quantities, provide unspecified health benefits to the host” [6, 7]. The majority of probiotics belongs to the lactic acid bacteria group [8], which includes *Lactococcus*, *Enterococcus*, *Pediococcus*, *Streptococcus*, and others.

Lactic acid bacteria affect host health through regulating immune system, increasing intestinal barrier function, maintaining intestinal flora balance, regulating nutrients and other mechanisms in animals [9]. Some lactic acid bacteria can increase the concentration of immunoglobulin G (IgG) and immunoglobulin M (IgM) and the activity of macrophages. IgG is the main component of serum antibodies, which has the functions of antibacterial, antiviral and prevention of infectious diseases. IgM, as the earliest antibody produced against pathogen invasion, has higher bactericidal activity and virus neutralization ability under the action of complement and phagocyte. Lactic acid bacteria regulate the host intestinal microflora by resisting the colonization of pathogenic bacteria. Different probiotic strains have specific adhesion regions, distinct immune effects, and varying modes of action [10]. Therefore, different Lactobacillus strains exhibit variations in their mechanism of action, and a particular strain may act through one or several mechanisms.

The effects of LAB on the growth performance, health status and meat quality of rabbits have been widely studied [11–14]. Most researchers have observed that the addition of LAB to rabbit diets can improve feed conversion and productivity, enhance host immune function and reduce morbidity and mortality. Nwachukwu [15] showed that incorporating lactobacilli and prebiotics into the diets of New Zealand rabbits was found to enhance their productive performance, intestinal development, and blood status, while also improving the digestion, absorption, and utilization of feed.

The complex microbiota of the rabbit gastrointestinal tract plays a key role in feed digestion, vitamin production, fermentation activity, stimulation of immune responses, production against pathogen infections and resistance to environmental stress. Due to the delicate nature of their digestive system and changes in gut microbiota, rabbits are prone to experiencing gastrointestinal disorders, such as acute or antibiotic-associated diarrhea [16, 17], during the feeding process.

One of the proposed mechanisms for the probiotic effect is through interaction with the host’s microbiota, such as by competitively preventing the colonization of pathogenic bacteria [18]. Another possible mechanism is the interaction of probiotics with the host immune system, leading to a modulation of the immune response [19].

In our study, we isolated lactic acid bacteria from the feces of healthy rabbits and evaluated their probiotics in vitro. *Enterococcus faecium* R1 and *Ligilactobacillus animalis* R2 have good effects in vitro antibacterial, and are resistant to acid and bile salt, and are sensitive to common antibiotics. However, their effects on preventing bacterial diarrhea in rabbits were still unknown. Therefore, the objective of this study was to investigate the effects of *Enterococcus faecium* R1 and *Ligilactobacillus animalis* R2 on diarrhea rates, immunoglobulin concentrations, short chain fatty acid profiles, and gut microbiota of rabbits during diarrhea, and thus to provide deeper insights of utilizing LAB to prevent bacterial diarrhea.

## Results

### Probiotic properties evaluation in vitro

**Fig. 1:**
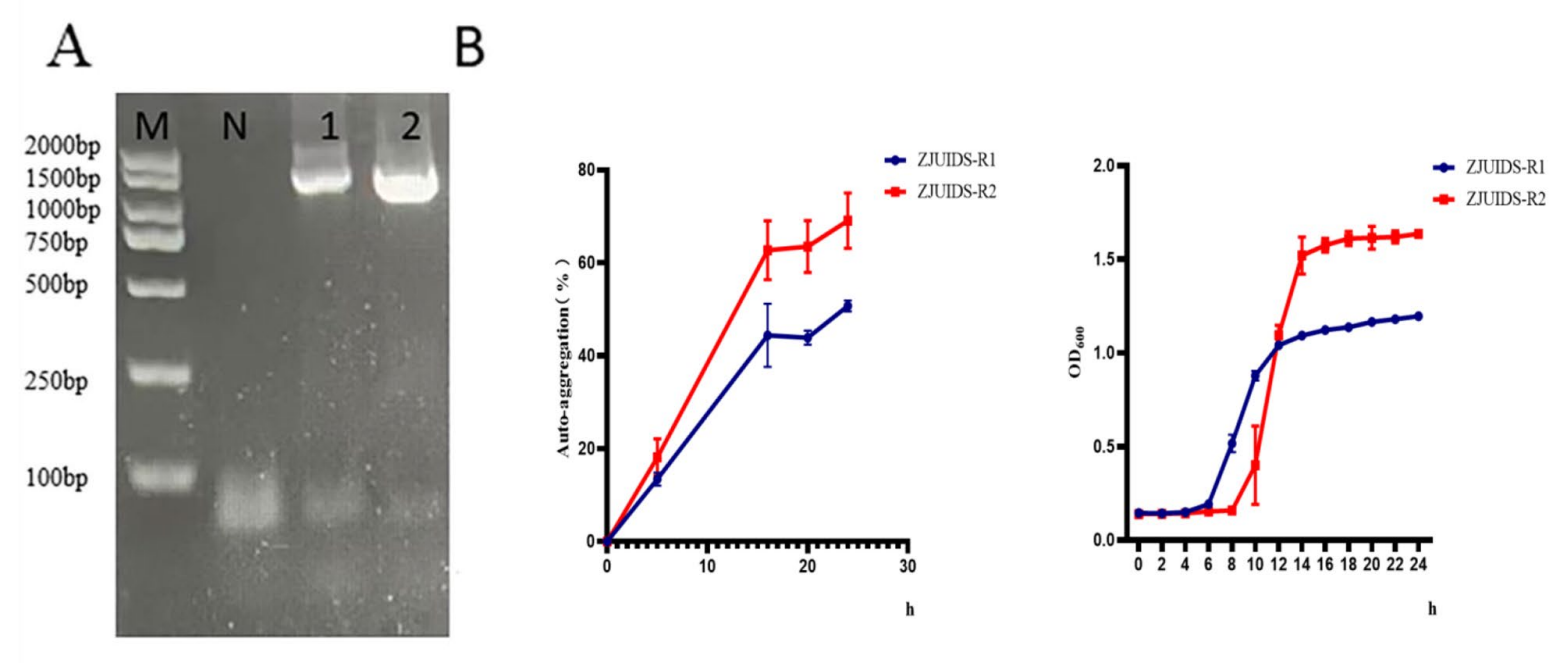
Identification and properties of lactic acid bacteria.(**A**) 16S rDNA amplification, 1: *Enterococcus faecium* ZJUIDS-R1; 2: *Ligilactobaciiius animalis* ZJUIDS-R2; N: Negative; M: 2000 bp DNA Marker; (**B**) Auto-aggregation (%) and Growth curve. The experiment was repeated three times and all data were expressed as means ± SD

A total of 97 suspected lactobacillus strains were screened from 53 fecal samples collected. According to the results of antibacterial activity, five strains were selected, among which ZJUIDS-R2 and ZJUIDS-R1 had the strongest antibacterial activity (Table 1). The results of 16 S rDNA identification showed that strain ZJUIDS-R1 was *Enterococcus faecium* and strain ZJUIDS-R2 was *Ligilactobacillus animalis* (Fig. 1A and Supplementary Fig. 1). The two strains exhibited promising probiotic properties in vitro, such as acid and bile salt tolerance, hydrophobicity and strong self-agglutination ability (Table 2; Fig. 1B). Both strains grew stably and were sensitive to common antibiotics used in the tests (Table 3).

**Table 1:** Antibacterial activity of LAB

		Inhibition zone diameters(mm)		
strains	Escherichia coli		Salmonella typhimurium	Staphylococcus aureus
H2	10.36 ± 0.30		16.03 ± 0.64^*^	9.52 ± 0.36^*^
H7	9.64 ± 0.35		17.16 ± 1.07^*^	8.31 ± 0.66
H35	9.31 ± 0.40		15.24 ± 4.47	9.34 ± 0.45
ZJUIDS-R1	11.30 ± 0.23^*^		17.21 ± 0.73^*^	10.63 ± 0.36^*^
ZJUIDS-R2	12.32 ± 0.18^*^		17.11 ± 1.34^*^	10.16 ± 0.63^**^

The experiment was repeated three times. All data were expressed as means ± SD. * *p* < 0.05; ** *p* < 0.01; *** *p* < 0.001

**Table 2:** survival rate and surface hydrophobicity

Strains	Survival rate of acid (pH = 1.5)	Survival rate of bile (0.3%)	Surface hydrophobicity (%)
ZJUIDS-R1	29.42 ± 6.37^*^	10.03 ± 2.67^*^	51.62 ± 1.52^*^
ZJUIDS-R2	20.91 ± 5.03	0.96 ± 0.25	29.83 ± 1.81

The experiment was repeated three times. All data were expressed as means ± SD. * *p* < 0.05; ** *p* < 0.01; *** *p* < 0.001

**Table 3:** Antibiotic sensitivity of LAB

Drug name	Paper content	Bacteriostatic zone diameter (mm)	
		ZJUIDS-R1 ZJUIDS-R2	
Penicillin	10 U	13.29 ± 0.73	19.42 ± 2.16
ampicillin	10 µg	16.14 ± 0.78	10.15 ± 1.62
gentamicin	30 µg	0	16.35 ± 1.17
tetracycline	10 µg	18.42 ± 0.56	11.27 ± 0.73
erythromycin	15 µg	6.75 ± 0.77	20.31 ± 0.22
ciprofloxacin	10 µg	11.53 ± 1.91	12.60 ± 1.51
chloramphenicol	30 µg	17.31 ± 1.91	22.16 ± 3.10

The experiment was repeated three times. All data were expressed as means ± SD

### Diarrhea Rates and Growth Performance

**Fig. 2:**
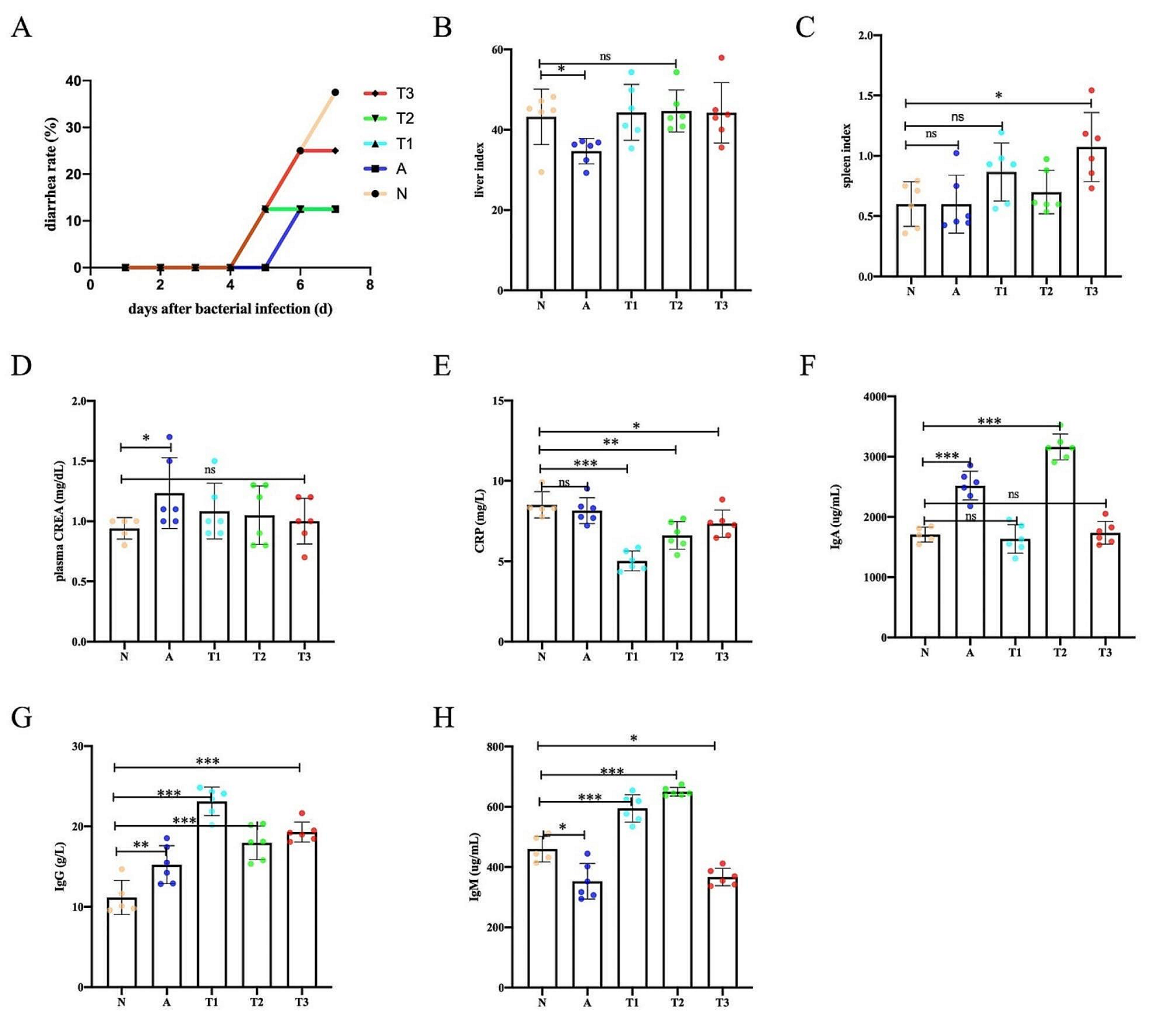
The effect of LAB on growth performance and blood parameters.(**A**) diarrhea rates; (**B**) liver index; (**C**) spleen index; (**D**) plasma CREA; (**E**) CRP level; (**F**) IgA level; (**G**) IgG level; and (**H**) IgM level. All data are expressed as means ± SD. * *p* < 0.05; ** *p* < 0.01; *** *p* < 0.001

4 days after the bacterial introduction, the rabbits began to develop diarrhea. The diarrhea rate of rabbits in the experiment is presented in Fig. 2A. In all of the groups, the group N had the highest incidence of diarrhea. The diarrhea rate of the T1, T2 and T3 groups were lower than the N group after the bacterial challenge. Meanwhile, the incidence of diarrhea was the same in A, T1 and T2 groups. As shown in Fig. 2B and C, the organ index of the liver and spleen kidney were summarized. The results showed that the liver index of the group A decreased compared to the other groups and the spleen index of the group T3 was significantly increased in weaning rabbits supplemented with *Enterococcus faecium* ZJUIDS-R1 and *Ligilactobaciiius animalis* ZJUIDS-R2 strains compared to the group N (*P*<0.05).

### Blood tests

As can be seen from Fig. 2D, the rabbits in A group showed a significant increase in plasma CREA levels compared to group N (*P*<0.05). No significant effects on other biochemical parameters were shown. According to Fig. 2E, rabbits in the T1, T2 and T3 groups exhibited decreased levels of CRP, compared to the group A rabbits (*P*<0.05). Both antibiotics and lactobacillus fed to weaned rabbits reduced indicators of systemic inflammation, with both strains of lactobacillus showing better reduction of systemic inflammation than antibiotics.

As shown in Fig. 2F, G and H, the concentration of the IgA of the group A and group T2 were significantly higher than group N (*P* < 0.001). Upon comparing the IgG antibody levels, it was observed that all four groups that were fed antibiotics and LAB showed significantly higher levels than the group N (*P* < 0.05), with the group T1, group T2, and group T3 displaying the greatest increase in antibody levels. IgM antibody concentrations in the T1 and T2 groups were significantly higher than the group N (*P* < 0.001), while the group A and group T3 had lower levels than the group N (*P* < 0.05).

### Effects of LAB on duodenum histology

**Fig. 3:**
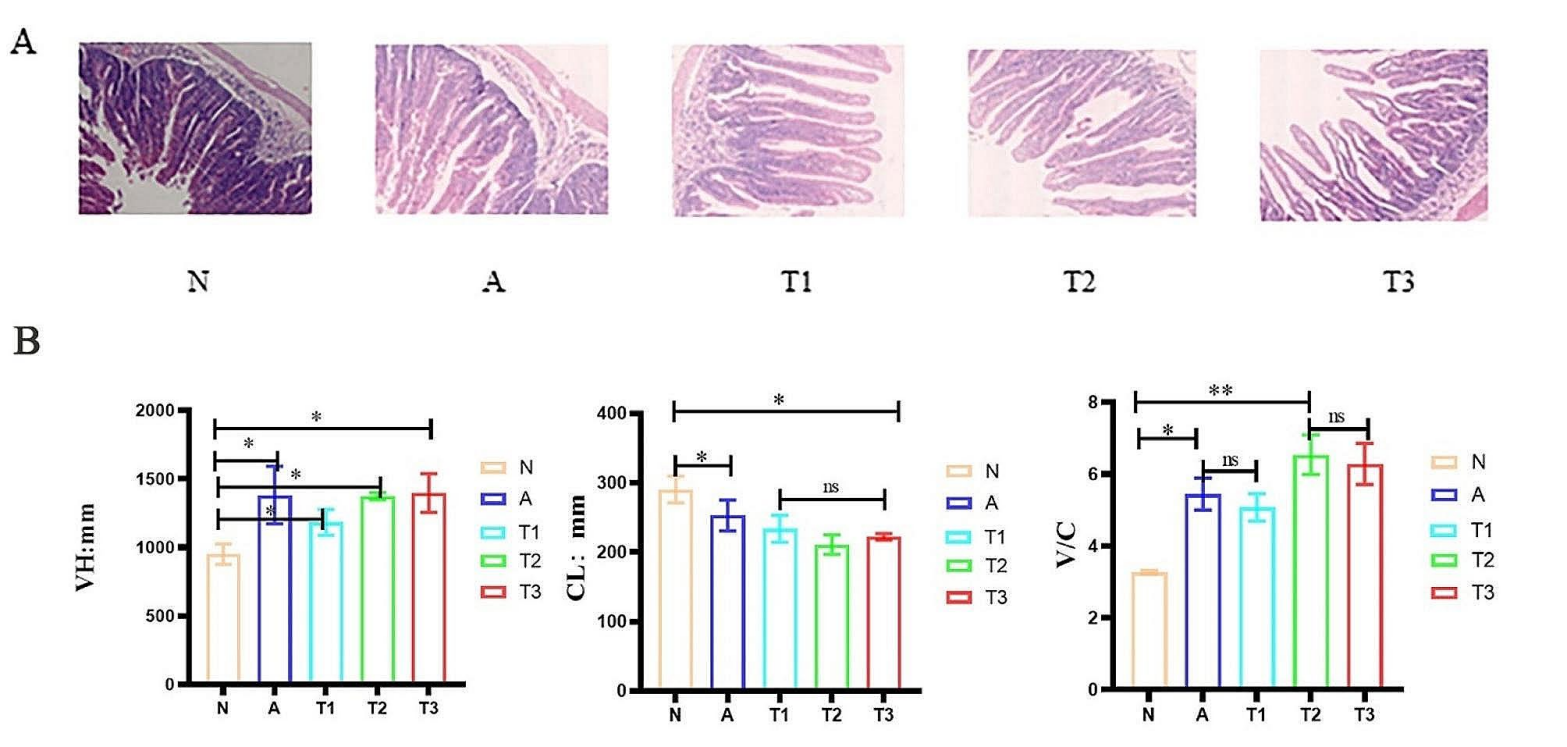
Enterococcus faecium R1 and Ligilactobacillus animalis R2 could alleviate duodenum intestinal morphological changes. (**A**) Duodenum H&E staining, magnification ×200; (**B**) Effect of LAB on duodenal length villous, intestinal crypt, and ratio of villous length to crypt depth. The experiment was repeated three times. All data were expressed as means ± SD. * *p* < 0.05; ** *p* < 0.01; *** *p* < 0.001. VH: villus height; CL: Crypt length; V/C: villus height/crypt length

During the necropsy after bacterial infection, duodenal effusion was observed, and the intestinal wall became thin and transparent (Fig. 3A). At the same time, the duodenum showed significant intestinal pathological changes, including villus atrophy and damage to the integrity of epithelial cells. Compared with group N, duodenal VH and V/C were significantly increased and CL was decreased in T1, T2 and T3 groups (*P* < 0.05) (Fig. 3B).These results indicated that these two strains could alleviate the morphological and pathological changes of the duodenum in young rabbits following bacterial infection.

### Effects of LAB treatment on SCFAs

**Fig. 4:**
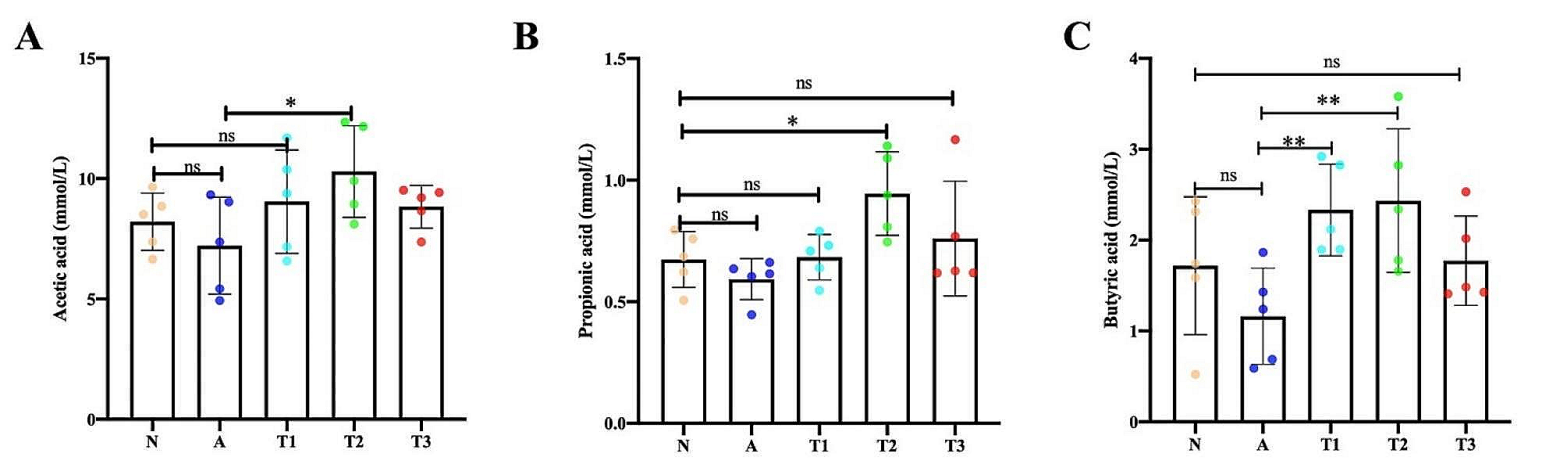
LAB administration increases the levels of short-chain fatty acids (SCFAs) in caecum contents. (**A**) Acetic acid; (**B**) Propionic acid; and (**C**) Butyric acid. All data are expressed as means ± SD. * *p* < 0.05; ** *p* < 0.01; *** *p* < 0.001

SCFA levels were measured in the cecum contents of each group. Group A had the lowest concentration of SCFAs among all groups, but there was no significant change compared to group N (Fig. 4A, B and C). The supplementation of *Enterococcus faecium* ZJUIDS-R1 to the rabbits’ diet resulted in a significant increase in the concentration of butyric acid (*P* < 0.05). Noticeable increase in the production of Propionic acid when rabbits were supplemented with *Ligilactobaciiius animalis* ZJUIDS-R2 (*P* < 0.05). However, there were no significant effects between the rabbits supplemented with *Enterococcus faecium* ZJUIDS-R1 and *Ligilactobaciiius animalis* ZJUIDS-R2.

### Effects of LAB treatment on gut microbiota

**Fig. 5:**
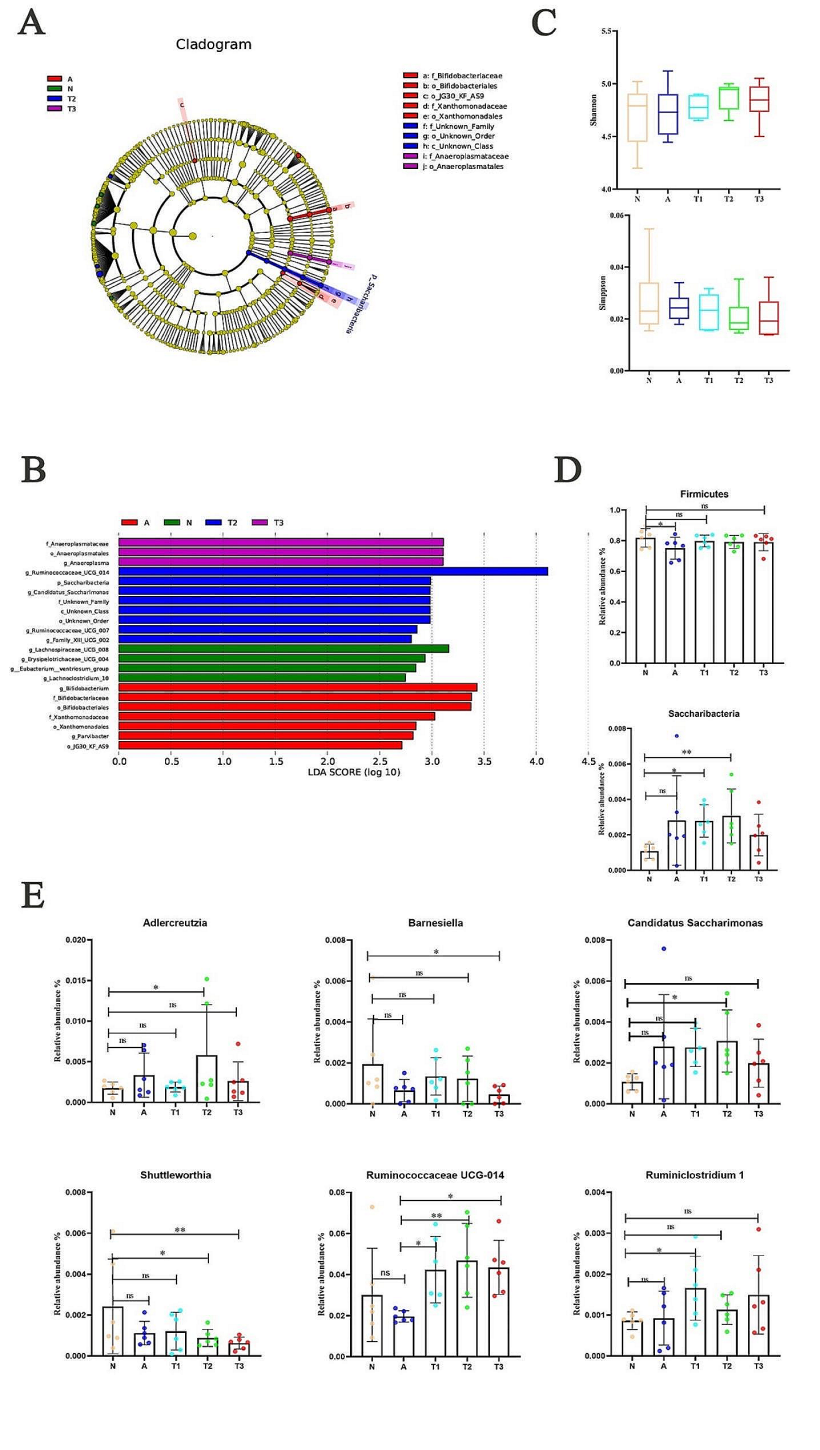
Enterococcus faecium ZJUIDS-R1 and Ligilactobacillus animalis ZJUIDS-R2 administration modify gut microbiota composition. (**A**) Taxa Lefse cladogram; (**B**) LDA Score; (**C**) Alpha-diversity; (**D**) Relative abundance of the fecal microbiota at the phylum level; (**F**) Relative abundance of the fecal microbiota at the genus level. All data are expressed as means ± SD. * *p* < 0.05; ** *p* < 0.01; *** *p* < 0.001

To investigate whether intestinal microorganisms are involved in the response of lactic acid bacteria to exposure to pathogenic intestinal bacteria, the microbiota composition was identified via 16 S rRNA gene amplicon pyrosequencing. Species taxonomic branching plots showing the stratified taxonomic distribution of significantly enriched species from the phylum to the genus level in each group of samples (Fig. 5A and Supplementary Fig. 2A). Distribution histograms reflecting LDA values for significantly different species, showing the significantly enriched taxa for each group and the identified underlying species (Fig. 5B and Supplementary Fig. 2B). In the results of the alpha diversity of cecal flora, there was no significant change in the Shannon index and Simpson index after supplementation with lactobacillus in weaned rabbits (Fig. 5C).

Firmicutes (72.5–82.1%) and Bacteroidetes (14.1–16.8%) were the major clades in all groups of the cecum, according to LEfSe analysis. As shown in Fig. 5D, antibiotics decreased the abundance of Firmicutes (*P* < 0.05). In contrast, there was a trend towards the increased abundance of weaned rabbits supplemented with antibiotics, *Enterococcus faecium* ZJUIDS-R1, and *Ligilactobaciiius animalis* ZJUIDS-R2 strains compared to the N group, though the change was not statistically significant.

Besides, both *Enterococcus faecium* ZJUIDS-R1 and *Ligilactobaciiius animalis* ZJUIDS-R2 alone can increase the abundance of Saccharibacteria (*P* < 0.05). At the genus level, *Enterococcus faecium* ZJUIDS-R1 increased the abundance of *Ruminiclostridium* 1, and *Ligilactobacillus animalis* ZJUIDS-R2 increased the abundance of *Adlercreutzia* and *Candidatus Saccharimonas*. *Enterococcus faecium* ZJUIDS-R1 and *Ligilactobacillus animalis* ZJUIDS-R2 can decrease the abundance of *Shuttleworthia* and *Barnesiella* (Fig. 5E).

### Discussion

The ideal lactobacillus strain should come from the specific animal species’ own gut, as this may lead to better compatibility and efficacy [20], which could avoid the effects of different gut animal-specific flora on the host, making it easier for the flora to colonize the host’s gut. The pH of the rabbit gastrointestinal tract is 2 ∼ 7, the pH of stomach cardia is 2 ∼ 3, and the pH of the small intestine is about 6 ∼ 7. The LAB in this experiment still maintained good tolerance under the acidic condition of pH 1.5, and were more likely to survive and colonize in the intestinal tract. It was found that the strains with high hydrophobicity had a strong binding ability to mucosal cells or a strong affinity for epithelial cells/mucus layer [21]. This suggests that after colonizing the host intestinal tract, the two strains could more effectively adhere to intestinal mucosal cells and exert immune regulatory effects.

Most studies [22, 23] have found that feeding lactic acid bacteria can effectively reduce the incidence of diarrhea in rabbits, which is consistent with the results of this study. In this study, the combined use of *Enterococcus faecium* ZJUIDS-R1 and *Ligilactobacillus animalis* ZJUIDS-R2 had a higher incidence of diarrhea than that used alone, which is consistent with the findings reported by A.y.e-Badaw [24, 25]. It is possible that the bacteriocins produced by the two strains in the intestine had an antagonistic effect, leading to the higher incidence of diarrhea.

Many studies [12, 15] have shown that LAB can digest and absorb nutrients in feed, and supplementation of LAB can improve the body weight and feed conversion rate of weaned young rabbits. In this study, no significant changes in rabbit growth performance occurred when rabbits were given 10^8^ CFU/kg of *Enterococcus faecium* ZJUIDS-R1 and *Ligilactobacillus animalis* ZJUIDS-R2, which was similar to the results of M.Pogany Simonova [25]. On the one hand, it is possible that the concentration of the bacterial solution was not sufficient, resulting in insufficient production of nutrients and digestive enzymes. On the other hand, it is possible that the concentration of the bacterial solution was too high and that too much *Enterococcus faecium* ZJUIDS-R1 and *Ligilactobacillus animalis* ZJUIDS-R2 were absorbing substances from the rabbits’ intestinal nutrition.

In similar studies, *Enterococcus faecium* added to adult horses significantly increased serum IgM antibody concentrations [26] while *Lactobacillus zeae* and *Lactobacillus casei* significantly increased IgM and IgG concentrations [27]. Previous research has demonstrated that administration of *Lactobacillus casei* SABA6 resulted in a significant increase in IL-6 and IL-10 concentrations, suggesting a potential protective effect on the host [23]. In this study, daily supplementation with *Enterococcus faecium* R1 and *Ligilactobacillus animalis* R2 significantly improved concentrations of the serum immunoglobulins IgA, IgG, and IgM, and decreased concentrations of the serum CRP ( *p* < 0.05 ). Therefore, it is inferred that *Enterococcus faecium* R1 and *Ligilactobacillus animalis* R2 might improve immune function.

In this study, it was found that the administration of *Enterococcus faecium* ZJUIDS-R1 and *Ligilactobacillus animalis* ZJUIDS-R2 could increase the duodenal villus height and villus-to-crypt ratio while decrease the crypt depth in weaned young rabbits (*P* < 0.05), indicating an improvement in intestinal development. It is suggested that *Enterococcus faecium* ZJUIDS-R1 and *Ligilactobacillus animalis* ZJUIDS-R2 positively impact the development of the duodenum in weaned young rabbits, but their effectiveness is not as comparable as that of antibiotics.

Rabbits belong to the category of hindgut fermenting animals, with the cecum containing the most abundant bacterial species [28], with about 10^10^∼10^12^ CFU/g microorganisms in its cecum. At the phylum level, Firmicutes was the main microorganism in intestinal tract [16, 29, 30], which can degrade plant cell wall components that cannot be decomposed by host digestive enzymes [31]. Most studies [29, 32] showed that in rabbits’ caecal flora, Firmicutes accounted for 75% ∼ 90%, and Bacteroidetes accounted for 10% ∼ 20%. In Crowley and Cotozzolo’s experiment [30, 33], a relationship of about 1:1 between Firmicutes and Bacteroidetes was found. It has been confirmed that at 14 days of age in young rabbits, Bacteroidetes are twice as abundant as Firmicutes, while Firmicutes will account for more than 90% of the cecal flora at 80 days of age in rabbits [34, 35]. Although there were differences in the proportion of Firmicutes and Bacteroidetes in different tests, there was no doubt that the two groups jointly dominated the population in the cecum, but the proportion of the two groups changed with the development of the life cycle of the organism.

In addition, these microorganisms are closely related to the host, and can participate in the body’s metabolism, promote host development, regulate the body’s immune function and prevent the invasion of pathogenic bacteria [11]. Bacteria in the intestine digest carbohydrates and proteins to produce a large amount of SCFA. SCFA in the cecum can reduce the pH in the intestine, reduce the growth of harmful bacteria [35], enhances intestinal epithelial barrier function, regulate host immunity, and reduce intestinal inflammation.

Consistent with previous studies, *Enterococcus faecium* R1 could significantly increase the concentration of butyric acid, and *Ligilactobacillus animalis* R2 could significantly increase the concentrations of acetic acid, propionic acid and butyric acid (*P* < 0.05). Our results indicate that *Ligilactobacillus animalis* R2 markedly increased the concentrations of SCFA, accompanied by up-regulation of the abundance of SCFA-producing bacteria, including *Ruminiclostridium* 1 and *Ruminococcaceae* UCG-014. *Ruminococcus* plays an important role in fiber digestion, digesting peptide sugars and cellulose and producing SCFA [16, 36], such as butyric acid. Butyric acid produced by intestinal microbial metabolism can induce intestinal barrier maturation [36], regulate energy and nutrient metabolism, and exhibit anti-inflammatory effects [37]. In this experiment, blood C-reactive protein concentrations in T1, T2 and T3 groups were significantly lower than group N, suggesting that the decrease in systemic inflammation of weaned young rabbits in these three groups may be caused by the increase of *Ruminococcus* in the intestinal tract and the increase of intestinal SCFA concentration. Overall, an increased abundance of SCFA-producing bacteria might be associated with increased SCFA, which is associated with improved systemic inflammation. Meanwhile, *Ligilactobacillus animalis* ZJUIDS-R2 can up-regulate the abundance of *Adlercreutzia*. *Isoflavones* produced by the metabolism of *Adlercreutzia* have antibacterial and anti-inflammatory effects.

*Enterococcus faecium* ZJUIDS-R1 and *Ligilactobacillus animalis* ZJUIDS-R2 can decrease the abundance of *Shuttleworthia* and *Barnesiella*. The genus *Sutterella* belongs to the family *Alcaligenaceae* within the b-Proteobacteria [38]. The increased abundance of Proteobacteria indicates the presence of an underlying biological disorder that predisposes animals to intestinal disease. *Sutterella* oversecretes IgA protease, which degrades IgA, thereby reducing IgA concentrations in the intestinal mucosa and impairing the function of the intestinal antimicrobial immune response [39–41]. It has been shown that pre-weaning diarrhoeic piglets significantly increase the relative abundance of *Prevotella*, *Sutterella* and *Anaerovibrio* fecal flora at the genus level.

In conclusion, supplementation with *Enterococcus faecium* ZJUIDS-R1 and *Ligilactobacillus animalis* ZJUIDS-R2 has been found to benefit the relief of diarrhea caused by bacterial infections in rabbits. This includes effects on reducing inflammation and intestinal damage. They can reduce intestinal damage, improve the body’s immunity, restore intestinal flora homeostasis and regulate intestinal short-chain fatty acid levels. This study provides valuable information for using *Enterococcus faecium* R1 and *Ligilactobacillus animalis* R2 as potential probiotics to prevent bacterial diarrhea in rabbits.

## Materials and methods

### Lactic acid bacteria and pathogen

Feces samples were collected from healthy rabbits at a flower and bird markets in Hangzhou, Zhejiang Province, China. To prepare the sample, one fecal pellet was mixed vigorously with I mL of sterile PBS, incubated for 10 min, and then centrifuged at 3000 × g for 5 min. After centrifugation, 100 µL of the supernatant was collected and subjected to gradient dilution with PBS. Several suitable dilution gradient were selected and coated on MRS Solid medium. After culture at 37 ℃ for 48 h, typical purified single colonies were selected and cultured in the sterile MRS Liquid medium at 37 ℃ for 24 h. After the culture, the lower layer of dense culture was mixed with sterile glycerin for preservation.

*Escherichia coli* ATCC 25,922 and *Salmonella typhimurium* ATCC 13,311 were purchased from the American Type Culture Collection. *Staphylococcus aureus* CMCC 26,003 was purchased from the Chinese Medical Bacterial Depository Management Centre.

### Screening for probiotic properties in vitro

#### Antimicrobial activities

The diffusion method was performed on agar using cultured broth. The indicator strains were revived and activated, and single colonies from the fourth round of activation were selected and inoculated into LB liquid medium. The bacterial cultures were incubated statically at 37 ℃ for 24 h, after which they were centrifuged at 8000 × g for 10 min. The prepared indicator bacteria were inoculated into LB solid medium (1% V/V), mixed well and poured into a plate with the Oxford cup.

After 2 h, the Oxford cup was carefully removed using sterile tweezers. Using enrofloxacin solution as the positive control, a total of 200 µL lactic acid bacteria cell suspension and fermentation supernatant were added to each well and cultured at 37 ℃ for 24 h. After culture, the diameter of the bacteriostatic zone was measured and recorded with a vernier caliper.

### 16 S rDNA amplification and sequencing

Bacterial genomic DNA was extracted by using a TIANamp Bacteria DNA kit (TIANGEN BIOTECH CO., LTD) following the manufacturer’s instructions and utilized as a template for PCR amplification with bacterial universal primers. The reaction was performed in a 32 µl volume containing 16 µl Mis, 2 µL nucleic acid template, 12 uL ddH2O and 2 uL the upstream and downstream primer mixture. Cycling conditions consisted of an initial predenaturation at 96°C for 5 minutes, followed by 35 cycles of denaturation at 96°C for 20 seconds, annealing at 62°C for 20 seconds, primer extension at 72°C for 20 seconds, and after the last cycle, the reaction is maintained at 72°C for 10 minutes. The forward primer was the following: 5’-AGAGTTTGATCCTGGCTCAG-3’, and the reverse primer was the following: 5’-TACGGCTACCTTGTTACGACTT-3’.The PCR products were recovered and sent to Sangon Bioengineering (Shanghai) Co., Ltd. for sequencing. The sequences were homologous compared in NCBI database (http://www.ncbi.nlm.nih.gov/).

### Acid tolerance and bile resistance

MRS broth was adjusted to pH 1.5 using HCl, and MRS was supplemented with 0.3% bile salt. The strain cultured was for 24 h and inoculated into the above MRS broth at 10% (v/v). The viable bacteria were counted with the method of plate culture count at 0 and 3 h, respectively. The survival rate was calculated using the following equation.

Survival rate (%) = A_3_/A_0_ × 100%.

A_0_ is the number of viable bacteria in the initial solution of the lactic acid strain and A_3_ is the number of viable bacteria of lactic acid bacteria strains treated with acid or bile for 3 h.

### Hydrophobicity assay

To the grown bacterial cultures (2 mL), 2 mL Xylenes was added and thoroughly mixed before incubating at 37 ℃ for 2 h. The optical absorbance of the aqueous phase was measured at 600 nanometers after 2 h. Percentage surface hydrophobicity or percent adhesion was calculated using the formula:

Surface hydrophobicity(%)=(A_0_ - A_2_)/A_2_ × 100%.

A_0_ is the initial OD and A_2_ is the OD at 2 h.

### Auto-aggregation assay

The overnight LAB culture, which had a bacterial count of 10^8^ CFU/mL, was harvested via centrifugation at 8000 × g for 15 min at 4 ℃. The bacteria were then washed three times with phosphate buffered salina. The washed cell pellet of LAB was suspended in Phosphate Buffer Salina until the absorbance of 0.5 ± 0.05 reached at 600 nm. The cell suspension was incubated for 24 h at 37 ℃. A 200 µL sample of the suspension was taken, and the absorbance was measured at 600 nm after 0, 2, 4, 6, 12, and 24 h. Percentage of auto-aggregation was calculated using the formula:

Auto-aggregation(%)=(A_0_ - A_t_)/A_t_×100%.

A_0_ is the initial OD and A_t_ is the OD at t hour.

### Growth curve assessment

Single colonies were inoculated into 45 mL MRS Medium and incubated at 37 ℃. Light absorption values at OD_600_ were measured every 2 h, and zero was used for blank MRS Culture. The growth curve of bacteria was plotted with culture time as the horizontal coordinate and light absorption value at OD_600_ as the vertical coordinate.

### Antibiotic susceptibility assessment

The disk diffusion method was used to measure the antibiotic sensitivity of lactic acid bacteria strains. The method of this study was referred to by the Institute of Clinical and Laboratory Standards Technical Guidelines (CLSI). The lactic acid bacteria suspension (10^8^ CFU/mL) was inoculated into MRS (1% V /V) solid medium, and mixed evenly to prepare MRS Plates. After the MRS plates were solidified, drug-sensitive papers were placed on plate. After culture at 37 ℃ for 24 h, the diameter of the antibacterial zone was measured with a vernier caliper and recorded.

### In vivo experiment

#### Animal trials

Four-week-old New Zealand rabbits were purchased from Shanghai SLAC Laboratory Animal Co. Ltd, Shanghai, China. A total of 40 weaned New Zealand Rabbits (Four-week-old, 8 per group) were used for the experiment, and every group was half males and half females. The New Zealand rabbits were housed in cages at 26 ± 2 ℃ and 50 ± 5% relative humidity with a 12-hour light/dark cycle. After acclimatization for 2 days, rabbits were randomly assigned to five groups: negative control group (group N, Phosphate buffer solution), antibiotic feeding group (group A, Enrofloxacin), treatment1 group (group T1, *Enterococcus faecium* ZJUIDS-R1), treatment2 group (group T2, *Ligilactobacillus animalis* ZJUIDS-R2), and treatment3 group (group T3, *Enterococcus faecium* ZJUIDS-R1 + *Ligilactobacillus animalis* ZJUIDS-R2).

During the first three weeks, all groups of rabbits were given a basic ration. Enrofloxacin was supplemented at a dosage of 3 mg/kg in the group A. *Enterococcus faecium* ZJUIDS-R1 + *Ligilactobacillus animalis* ZJUIDS-R2 were supplemented at a dosage of 10^8^ CFU/kg/day in the group T1 and group T2, separately. *Enterococcus faecium* ZJUIDS-R1 + *Ligilactobacillus animalis* ZJUIDS-R2 were supplemented at a dosage of 0.5*10^8^ CFU/kg/day in group T3. In the fourth week, *Escherichia coli*, *Salmonella typhimurium* and *Staphylococcus aureus* were supplemented at a dosage of 10^8^ CFU/kg/day in all groups. Food intake was measured daily, and the amount of food given to each group was recorded. All procedures were approved by the Animal Care and Use Committee of the Laboratory Animal Center of Zhejiang University (ZJU20230211).

### Sample collection

Each young rabbit underwent intravenous blood collection. At the end of the experiment, all animals were euthanized. The procedure was as follows: firstly, isoproterenol (6 mg/kg) was injected intravenously at the ear margin for sedation, followed by 500 ml of air to cause death. Internal organs, each section of bowel and each section of bowel contents were dissected. The liver, heart, lung, kidney, and pancreas were separated and weighed. All the collected samples were immediately transferred into liquid nitrogen for temporary storage and finally stored at − 80 °C for further analysis.

### Diarrhea Rates and Growth Performance

Body weight and feed consumption were measured every week during the experiment; average daily weight gain and feed conversion were calculated mathematically. Diarrhea rate was also recorded in groups daily after feeding a large number of pathogenic bacteria. The incidence of diarrhea was calculated as follows: diarrhea incidence (%) = [(number of rabbits with diarrhea) / (number of rabbits × experimental days)] × 100%. The organ index was calculated as follows (%) = [(organ weight) / (body weight before death)] × 100%.

### Biochemical analyses

Plasma was prepared by centrifugation of blood in sodium heparin collection tubes at 8000 r/min for 2 min. Plasma glucose (GLU), blood creatinine (CREA), blood urea nitrogen (BUN), total protein (TP), albumin (ALB), globulin (GLOB), albumin-to-globulin ratio (ALB/GLOB), alanine aminotransferase (ALT) and alkaline phosphatase (ALKP) were measured using an IDEXX automatic biochemistry instrument.

Plasma levels of Immunoglobulin A (IgA), Immunoglobulin G (IgG), Immunoglobulin M (IgM), and C-reaction protein (CRP) were determined by ELISA determination kits (Hangzhou Hannuo bio Co., Hangzhou, China).

### Duodenum histological analysis

After the experiment, the abdominal cavity of weaned young rabbits was opened after death, and all parts of the intestine were separated gently. Duodenum samples were selected from the same location without squeezing. Duodenum samples were collected from a location 4 cm after the stomach was connected to the duodenum, and the length of the sample was about 1 ± 0.5 cm. The duodenum tissues were fixed in 4% paraformaldehyde for 24 h. The duodenum tissues were embedded in paraffin and cut into 4-µm sections, which were then stained with hematoxylin and eosin (H&E) and analyzed under light microscopy.

To analyze the effects of LAB on the intestinal morphology of weaned rabbits, the villus height, crypt depth and crypt ratio of duodenum sections were observed.

### Determination of SCFA levels in fecal samples

The fecal samples were diluted three-fold with ultrapure water and vortexed for 10 min. The suspension was centrifuged at 4 ℃, 10 000 × g for 10 min, and the supernatant was collected. The supernatant was then centrifuged at 4 ℃, 10 000 × g for an additional 10 min. One milliliter of supernatant with 20 µL of chromatographic grade phosphoric acid (Shanghai Aladdin Biochemical Technology Co. Ltd.), and the mixture was passed through a 0.22 μm disposable syringe filter and the filtrate was injected directly into the chromatography vial (Wonda Vial, Shimadzu, Corp., Kyoto, Japan). The content of SCFAs (mainly acetic, propionic and butyric acids) in the contents of the caecum was determined using a gas chromatograph (Shimadzu, Corp., Kyoto, Japan), as previously described [42].

### 16S rRNA gene sequencing

The caecum samples were sent to Hangzhou MKbio for total DNA extraction, followed by 16S rRNA gene high-throughput sequencing technology. The V3-V4 region of the bacteria 16S ribosomal RNA gene was amplified by PCR using primers 341 F (5ʹ-barcode- CCTAYGGGRBGCASCAG)-3ʹ and 806 R (5ʹ-GGACTACHVGGGTWTCTAAT-3ʹ), where the barcode is an eight-base sequence unique to each sample. The sequencing was conducted by an Illumina Novaseq platform (PE300, San Dego). Raw sequences were subjected to a quality-control process via UPARSE. Sequences with ≥ 97% similarity were assigned to the same Operational Taxonomic Units (OTUs) using USEARCH. The 16S rRNA gene data were also submitted to the GEO repository.

### Statistical analyses

The analyses were performed using Graph Pad Prism (Graph Pad software 8.2.1). Date are expressed as the means ± standard deviation (SD). Results were analyzed using one-way analysis of variance (ANOVA) with SPSS software 23.0 (SPSS Inc., Chicago, Illinois, USA). In all tests, *P* values of < 0.05 were considered statistically significant.

### Electronic supplementary material

Below is the link to the electronic supplementary material.

Supplementary Material 1

## Data Availability

The nucleotide sequences have been stored in a database, and the relevant accession numbers are OR544987 and OR544988. The datasets generated and/or analyzed during the current study are available from the corresponding author on reasonable request.
