# Supplementary material for: Lactic acid bacteria reduce bacterial diarrhea in rabbits via enhancing immune function and restoring intestinal microbiota homeostasis
Source: BMC Vet Res. 2024 Apr 20;20:151. doi: 10.1186/s12917-024-03981-5 (PMC11031951; doi:10.1186/s12917-024-03981-5)
Supplement: Supplementary file 1 — Supplementary Material 1 [file 12917_2024_3981_MOESM1_ESM.pdf]

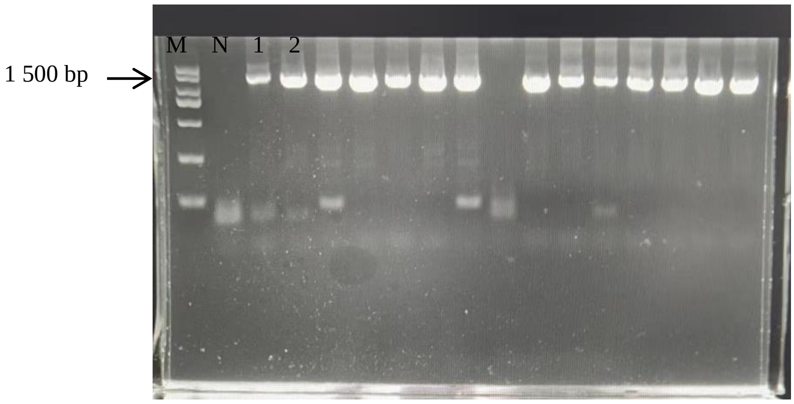

Supplementary figure 1 16S rDNA amplification

1：*Enterococcus faecium* ZJUIDS-R1；2：*Ligilactobaciiius animalis* ZJUIDS-R2；N：Negative；M： 2000bp DNA Marker;

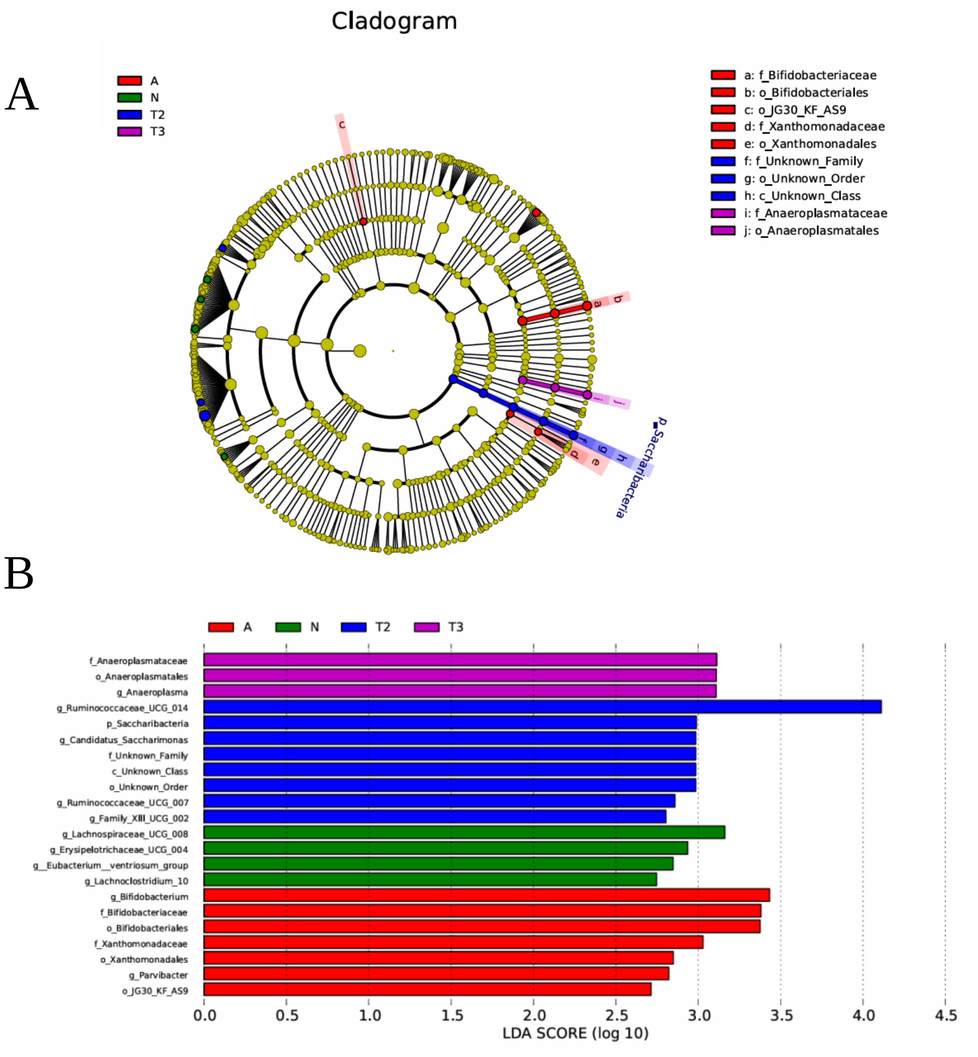

Supplementary figure 2 Enterococcus faecium ZJUIDS-R1 and Ligilactobacillus animalis ZJUIDS-R2 administration modify gut microbiota composition.

(A) Taxa Lefse cladogram; (B) LDA Score;
